# Characterization of tongue worms, *Linguatula* spp. (Pentastomida) in Romania, with the first record of an unknown adult *Linguatula* from roe deer (*Capreolus capreolus* Linnaeus)

**DOI:** 10.1007/s00436-022-07566-9

**Published:** 2022-06-11

**Authors:** Diane P. Barton, Calin Mircea Gherman, Xiaocheng Zhu, Shokoofeh Shamsi

**Affiliations:** 1 School of Agricultural, Environmental and Veterinary Sciences, Charles Sturt University, Wagga Wagga, NSW Australia; 2 University of Agricultural Sciences and Veterinary Medicine Cluj-Napoca, Calea Manastur Street, 3–5, Cluj-Napoca-Napoca, Romania; 3 NSW Department of Primary Industries, Wagga Wagga Agricultural Institute, Wagga Wagga, NSW Australia

**Keywords:** Introduced species, Biosecurity, Veterinary, Wildlife, Companion animals

## Abstract

Specimens of the pentastomid parasite, *Linguatula serrata*, have been reported from several animals in Romania, including some domestic dogs translocated to other parts of Europe. In this study, gray wolves (*Canis lupus*, *n* = 80), golden jackals (*C. aureus*, *n* = 115), red foxes (*Vulpes vulpes*, *n* = 236), and roe deer (*Capreolus capreolus*, *n* = 1) were examined for pentastomes. Overall, 17.5% of wolves were found to be infected with specimens of *Linguatula*, with a range of infections of one to five individuals per animal. Golden jackals and foxes had much lower infection levels, with 1.73% of golden jackals and 1.69% of foxes infected; both host species were found to be infected with one or two individual pentastomes per animal. The single deer specimen was infected with three individual pentastomes. The pentastomes collected from the wolves and golden jackals were determined to be immature and mature adult specimens of *L. serrata* based on morphological examination and molecular analysis using the 18S rRNA gene. No pentastomes collected from the red foxes were available for identification. The pentastomes collected from the roe deer were expected to be *L. arctica* but determined to be mature adult male specimens of an unknown *Linguatula,* herein, referred to as *Linguatula* sp. based on its morphology; the results of molecular sequencing for the *Linguatula* specimen collected from the deer were inconclusive, preventing a final species identification. This study presents the first report of *L. serrata* in any hosts from Romania through both morphological and molecular characterization, and also presents the first report of a *Linguatula* sp. in *Ca. capreolus*, utilizing morphological characterization. Issues of morphological variability are discussed, including the presence of spines in the hook pit of specimens of *Linguatula*. This study highlights the need to examine all specimens of *Linguatula* to confirm the stage of development. Despite the inconclusive molecular result for some specimens, the authors still urge future researchers to incorporate a combined molecular and morphological approach in identifying specimens of *Linguatula*.

## Introduction 

Members of the pentastomid genus *Linguatula* Frölich, 1789 have a long and problematic taxonomic history (Gjerde 2013). Adult *Linguatula* are generally found in the nasal cavities of carnivorous definitive hosts, such as wolves, dogs, and foxes, with nymphal stages present in various organs of herbivorous animals (Shamsi et al. 2017b; Barton et al. 2019, 2020).

As with many other genera of pentastomids, identification to genus level is generally certain, but identification to species level “remains abstruse” (Kelehear et al. 2011). This taxonomic confusion has been compounded by the identification of any pentastomid removed from the nasal sinuses of a mammal as *Linguatula serrata* (Frölich 1789) without morphological verification (Riley et al. 1987). Indeed, adult *Linguatula* were commonly reported in the nasal passages of reindeer (*Rangifer tarandus* (Linnaeus)) in Norway and were immediately referred to as *L. serrata*, the cosmopolitan species, until Riley et al. (1987) determined them to be *L. arctica* Riley et al., 1987, a reindeer-specific parasite with a postulated direct life cycle (Haugerud and Nilssen 1990). The use of molecular characterization will assist in species identification (Kelehear et al. 2011); however, without proper taxonomic identification through morphological characteristics of the specimen/s first, it will only aid in cementing the taxonomic irregularities.

A survey of various animals in Romania identified a number of specimens of *Linguatula*. There are a number of previous reports of *L. serrata* from animals in Romania (Gherman et al. 2002; Negrea et al. 2009; Ioniță and Mitrea 2016), including in dogs exported from Romania (Villedieu et al. 2017; Wright et al. 2020; Berberich et al. 2022); however, there has been no combined morphological or molecular characterization of the specimens. Gjerde (2013) and (Berberich et al. 2022) molecularly characterized specimens of *L. serrata* from a dog from Romania imported to Norway and Germany, respectively, but without morphological characterization. Thus, this study aims to provide the first combined morphological and molecular characterization of specimens of *Linguatula* collected from various hosts in Romania.

## Materials and methods

### Parasites

The following hosts were examined for pentastomes: gray wolves (*Canis lupus* Linnaeus, 1758) (*n* = 80) (collected 1998–2010), golden jackals (*C. aureus* Linnaeus, 1758) (*n* = 115), red foxes (*Vulpes vulpes* (Linnaeus, 1758)) (*n* = 236), and roe deer (*Capreolus capreolus* (Linnaeus, 1758)) (*n* = 1) (all collected from 1998). All animals were lawfully collected by local hunters in accordance with the relevant guidelines and regulations and the carcasses were made available for dissection. The nasal cavities of the various carnivores could not be accessed by the method utilized in Shamsi et al. (2017b). Instead, the palatine mucosa was removed which exposed the sphenopalatine foramen; a hose was passed through the foramen from the buccal cavity onto the nasal cavity and water flushed the nasal cavity with enough pressure to eliminate the *Linguatula* specimens through the nostrils. The internal organs of the deer were examined following standard parasitological examination techniques (https://www.agric.wa.gov.au/livestock-biosecurity/ruminant-animal-post-mortem-guide?page=0%2C1); the head was not examined.

Most pentastomes were preserved in ethanol (approximately 99.3%) for further molecular and morphological examination; one specimen was preserved in 10% buffered neutral formalin for morphological examination only.

**Fig. 1:**
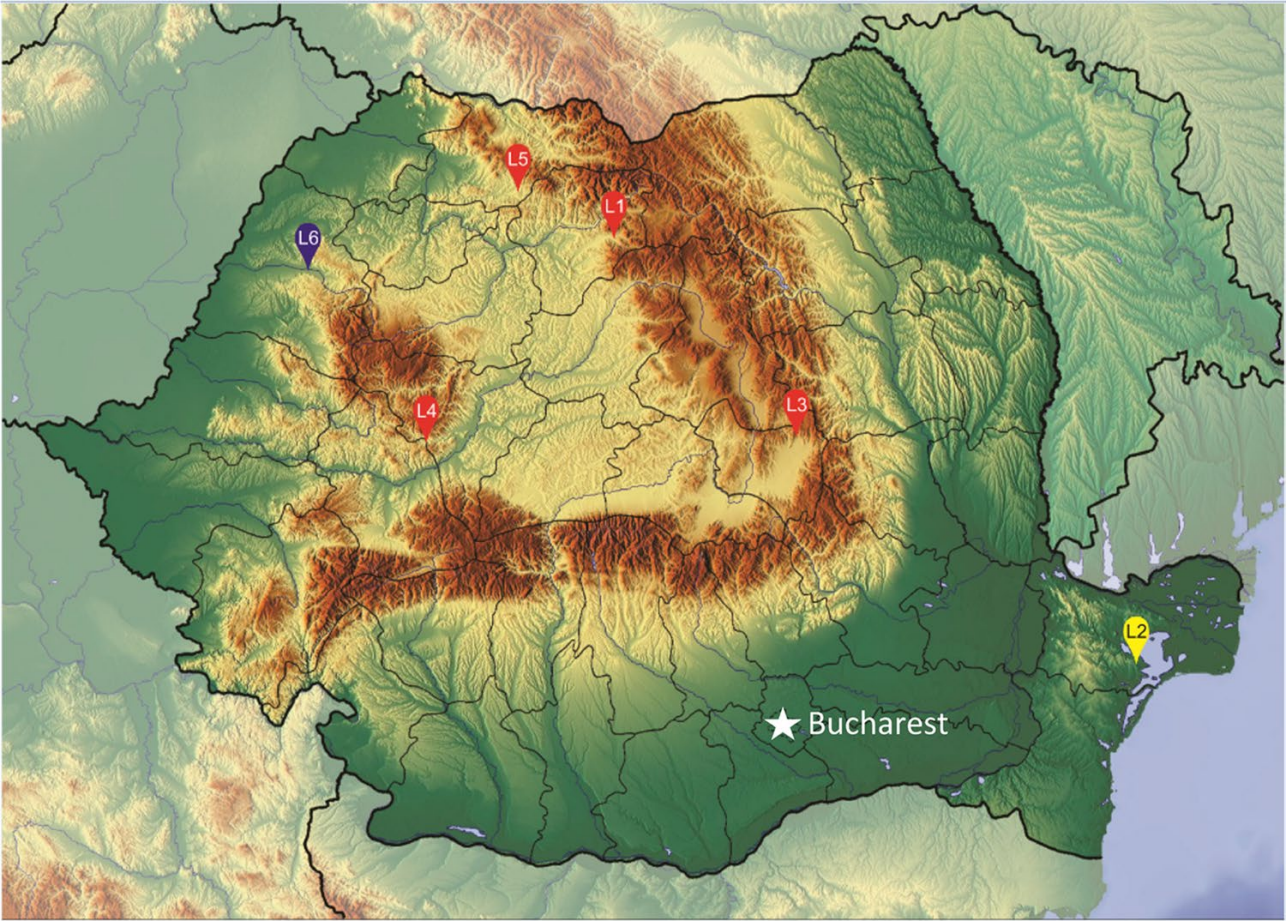
Map of the collection locations for the pentastomes examined in this study. Locations are indicated by red (collected from *Canis lupus*), yellow (collected from *Canis aureus*), and blue (collected from *Capreolus capreolus*) markers. The number within the marker refers to the specimen listed in Table 1

Eight pentastome specimens were sent to the Parasitology Laboratory, Charles Sturt University, Australia, for identification. Collection details of these specimens are provided in Table 1 and Fig. 1.

**Table 1:** Collection information for the specimens of *Linguatula* Frölich, 1789 collected from wild carnivores and ungulates in Romania that were sent to Shamsi’s Parasitology Laboratory, Charles Sturt University, Australia (SPL), for identification. Specimens have been deposited in the Australian Museum (AM) collection. Genetic sequences have been deposited in GenBank

Collection code	SPL code	Origin			Parasite life stage	Site in host	AM number	GenBank number
		Host species	County	Locality				
L1	1098	*Canis lupus*	Bistrita Bargaului	Bistrita Nasaud	Mature female	Nasal cavity	TBA	TBA
L3	1164	*Canis lupus*	Catrusa	Covasna	Mature male	Nasal cavity	TBA	N/A
L4	–	*Canis lupus*	Salistea	Alba	Mature male	Bronchial tree	N/A	N/A
L5	–	*Canis lupus*	Valea Chioarului	Maramures	Mature female	Nasal cavity	N/A	N/A
L2	1106	*Canis aureus*	Salcioara	Tulcea	Immature female	Nasal cavity	TBA	N/A
L6	1165	*Capreolus capreolus*	Abram	Bihor	Mature males	Lung tissue	TBA	TBA

### Morphological examination

#### Light microscopy

Important characteristics of pentastomes’ features were studied/measured (in mm). For details see Shamsi et al. (2020a) and Barton et al. (2020). In brief, the number of annuli was counted. One set of hooks and the oral cadre were dissected from each specimen, mounted in lactophenol, and observed using light microscopy. Hook dimensions measured for all specimens were blade length (AC), hook length (AD), base length (BC), plateau length (CD), and hook gape (AB) (Shamsi et al. 2020a). Buccal cadre measurements were of buccal cadre length and buccal cadre width. For male specimens, the length and width of the copulatory spicule were measured. Photos of slide mounted specimens were taken using a 9-MP microscope digital camera (AmScope Model MU900).

#### Scanning electron microscopy (SEM)

Four pentastome specimens were selected for scanning electron microscopy (SEM), including two from wolves (one adult female and one adult male) and two from deer (both males). The female specimen was initially preserved in formalin, while all male specimens were initially preserved in ethanol. To obtain SEM images, the specimens were washed in 70% ethanol and dehydrated using a graded series of ethanol. Excess 70% ethanol was removed from around the sample using a transfer pipette before being replaced by 80% ethanol overnight. This step was repeated with 90%, 95%, and 100% ethanol and followed by 100% anhydrous ethanol dehydration three times. The sample was then dried in liquid CO_2_ in a Tousimis® Autosamdri-931 critical point dryer (USA). A carbon tab was used to attach the sample to a stub before gold coating (25 mA for 2 min) in an Emitech K550X Sputter Coater (Quorum Technologies, UK). The sample was examined in a scanning electron microscope (JCM-5000 Benchtop SEM NeoScope, Jeol Ltd, USA) with accelerating voltage set at 10–15 kV.

### DNA extraction, PCR, and sequencing

A small piece of tissue was collected from each of two pentastome specimens for genomic DNA extractions: one collected from a wolf (SPL 1098) and one from a deer (SPL 1165). Molecular analysis was completed using DNeasy Blood & Tissue Kits (Qiagen, Australia) according to the modified version of the manufacturer’s protocol after elution into 45 μl of water (Shamsi et al. 2017a). The cytochrome oxidase subunit 1 (Cox1) gene and 18S ribosomal RNA (18S) gene were amplified using the primer sets and PCR conditions as described by Gjerde (2013). PCR amplicons were sent to the Australian Genome Research Facility (Queensland) for bidirectional sequencing using the same primers as the PCR. Forward and reverse AB1 trace files were quality checked using Seq Scanner (Applied Biosystems/Thermo Fisher). Sequences of the 18S region were aligned with the Geneious alignment algorithm (Geneious version 11.1.4) (Kearse et al. 2012), and then were double checked with all variable sites in the original trace files for confirmation. Alignments were then truncated to 1751 bp, based on the shortest sequence.

## Results

Overall, 14 (17.5%) wolves were found to be infected with specimens of *Linguatula*, with a range of infections of one to five individuals per animal. Golden jackals and foxes had much lower infection levels, with two (1.73%) golden jackals and four (1.69%) foxes infected; both species were found to be infected with one or two individuals per animal. The single deer specimen was infected with three individuals.

### Morphological results

Of the specimens sent to Australia, the specimens collected from wolves and the golden jackal were determined to be immature and mature adult specimens of *L. serrata* based on morphological and molecular analysis. No specimens collected from the red fox were sent for identification. Measurements of individual specimens are provided in Table 2. These measurements are compared to specimens identified as *L. serrata* collected from wild dogs in Australia. The specimen from the wolf was a mature adult female, whereas the specimen from the golden jackal was an immature adult female. Both specimens had much larger hook measurements than those presented by Shamsi et al. (2020a) for *L. serrata* collected from wild dogs and foxes in Australia.

**Table 2:** Morphological measurements of individual specimens of *Linguatula serrata* (Frölich 1789) collected from *Canis lupus* Linnaeus and *C. aureus* Linnaeus from Romania in this study compared to specimens from *C. familiaris* Linnaeus in the literature. Measurements are presented in µm

SPL specimen number	1098	1106		1164	
Characteristic	Female	Female (immature)	Female	Male	Male
Host species	*Canis lupus*	*Canis aureus*	*Canis familiaris*	*Canis lupus*	*Canis familiaris*
Total length	9000	2450	5990 (4800–7000)	2000	1640 (1500–1800)
Max width	1000	500	790 (700–850)	350	320 (250–400)
No. annuli	90	–	92 (86–109)	83	80 (75–89)
Anterior hook					
AC	290	–	160.6 (145–175)	–	129.6 (113–150)
AD	550	–	252.5 (235–280)	–	211 (195–235)
BC	280	240	142.5 (105–165)	175	101.7 (90–115)
CD	330	290	132.5 (105–165)	285	100.8 (75–130)
AB	200	–	92 (75–105)	–	82.6 (75–100)
FL	1000	–	510 (500–520)	650	265
Posterior hook					
AC	310	–	150 (140–160)	–	136 (120–150)
AD	580	–	270 (250–290)	–	212 (200–230)
BC	310	235	145 (110–160)	165	108.3 (100–120)
CD	310	–	145 (110–180)	285	103.3 (90–130)
AB	200	–	100 (90–110)	–	94 (80–130)
FL	–	–	470	–	145 (130–160)
Buccal cadre L	700	570	227.5 (130–300)	360	180
Buccal cadre W	590	410	197.5 (90–270)	315	180
Copulatory spicule L				937.5 (875–1000)	448.5 (430–460)
Copulatory spicule W				355 (350–360)	162.5 (140–175)
Geographical location	Romania	Romania	Australia	Romania	Australia
Reference	This study	This study	Shamsi et al. (2020a)	This study	Shamsi et al. (2020a)

**Fig. 2:**
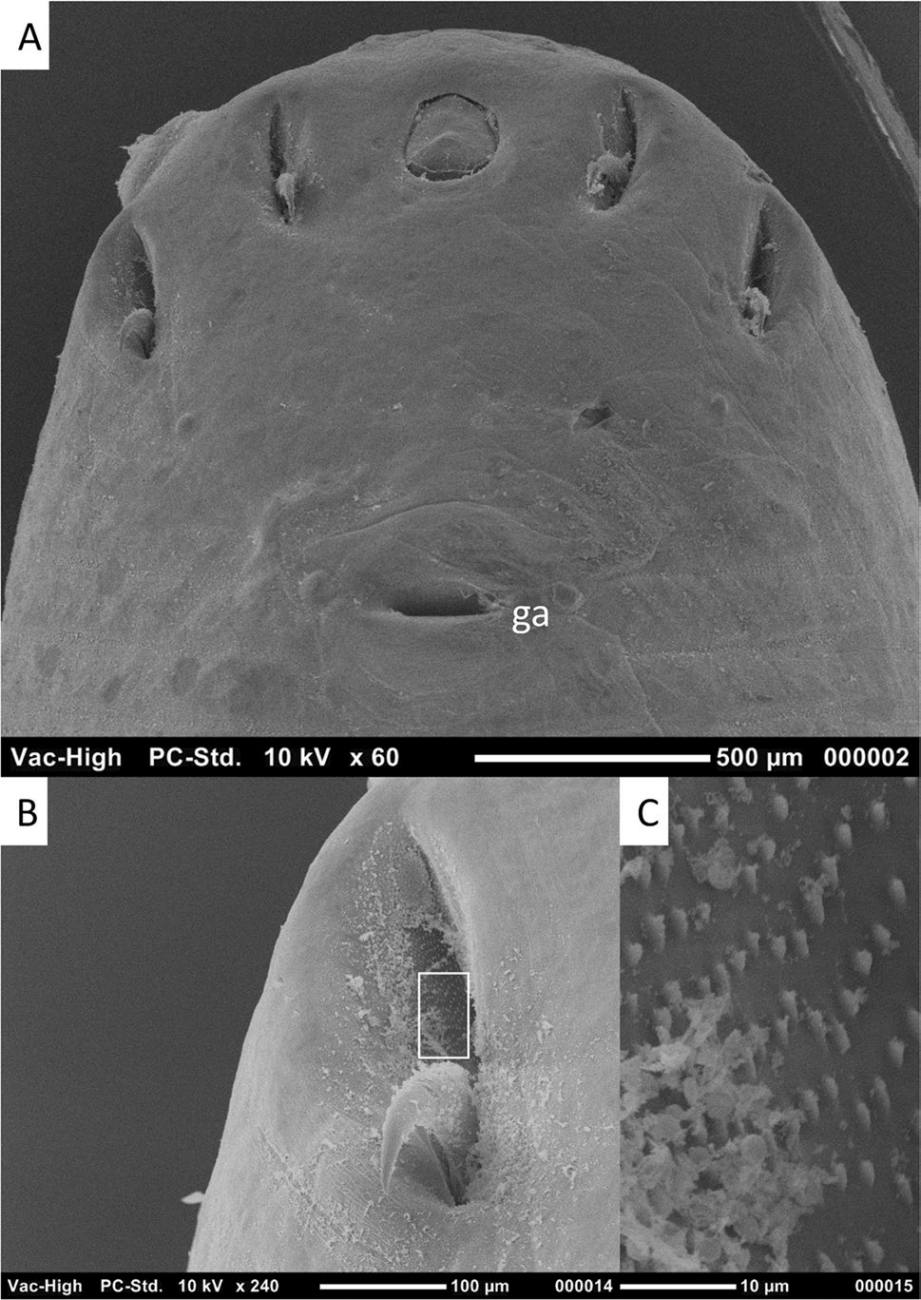
Scanning electron microscopy images of a male specimen of *Linguatula serrata* (Frölich 1789) collected from *Canis lupus* (specimen number L4). **A** Anterior end of specimen, ventral view; ga, genital atrium. **B** Posterior hook with hook “pit” showing spines on wall (white box). **C** Spines on wall of hook “pit”

SEM of the male specimen of *L. serrata*, collected from a wolf (Fig. 2), showed the wall of the posterior hook pit to be lined with fine spines. The spines did not appear to be present on the base of the hook itself.

**Fig. 3:**
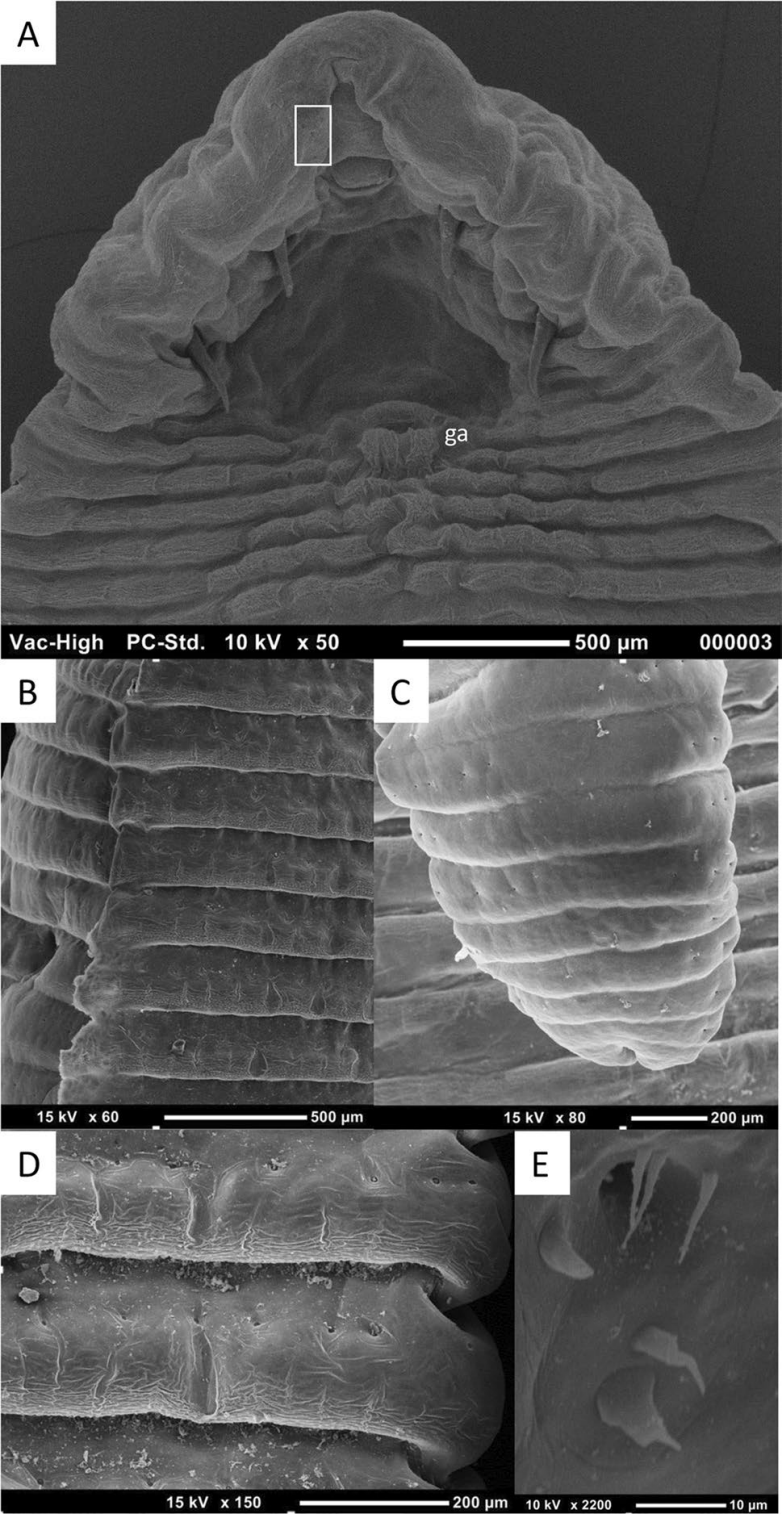
Scanning electron microscopy images of male specimens of *Linguatula * sp. collected from *Capreolus capreolus*. **A** Anterior end, ventral view (SPL1165-3), white box marks location of sensory sensillae presented in **E**; ga, genital atrium. **B** Lateral edge of body (SPL1165-2), ventral side to the right. **C** Posterior end of body, dorsal view (SPL1165-2), note absence of chloride cells in midline of dorsal side. **D** Abdominal annulus, ventral view (SPL1165-2), note single row of chloride cells and markings on posterior edge of annuli. **E** Sensory pore, ventral surface of anterior end (SPL1165-3)

**Fig. 4:**
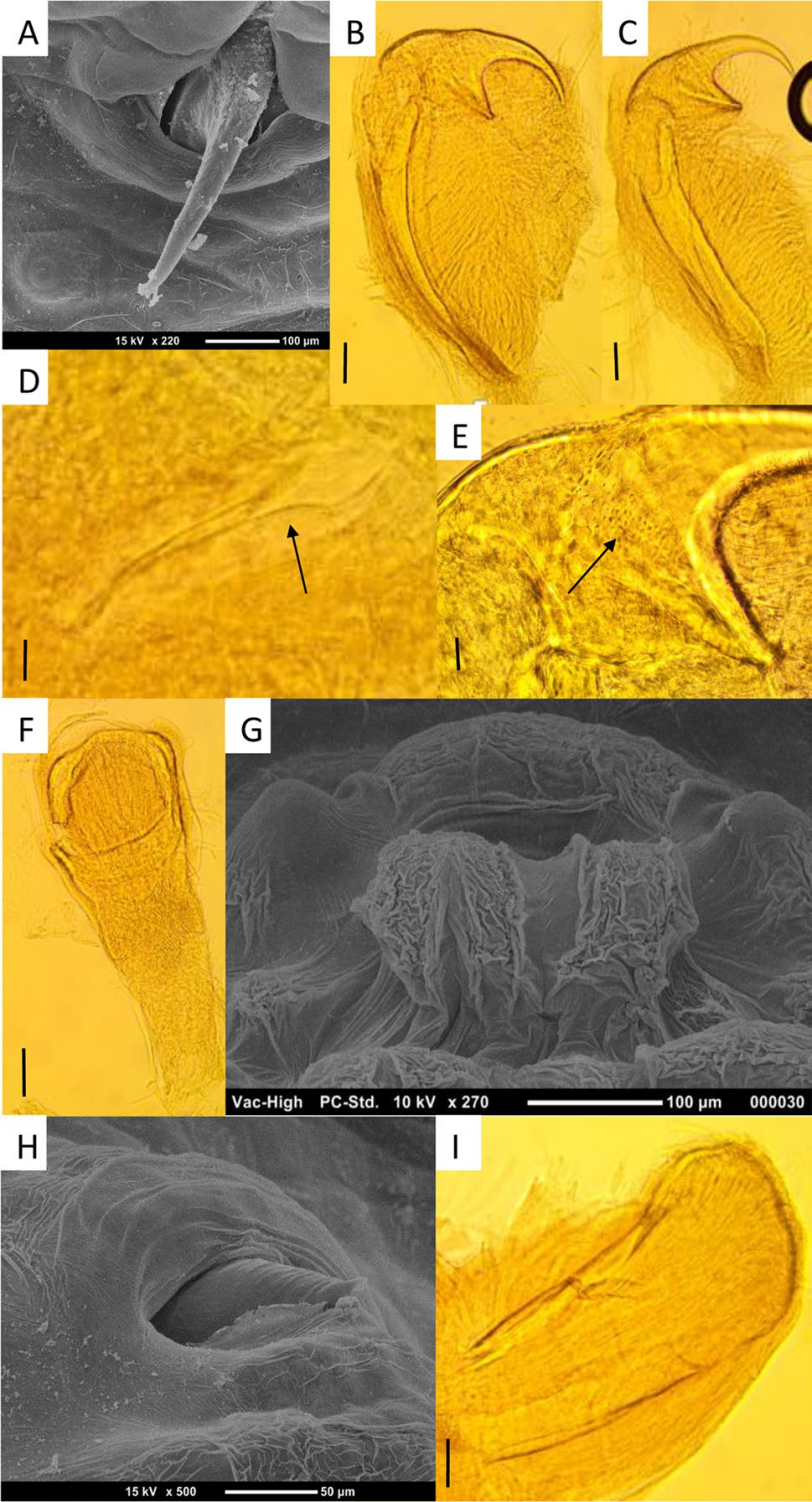
Light and scanning electron microscopy images of male specimens of *Linguatula * sp. collected from *Capreolus capreolus*. **A** SEM of posterior hook (SPL1165-2), with sensory papillae #3. **B** Anterior hook with fulcrum (SPL1165-2). Scale bar = 100 µm. **C** Posterior hook with fulcrum (SPL1165-2). Scale bar = 100 µm. **D** Posterior hook opening “pit” (SPL1165-2), showing fine spines on cuticle (arrow). Scale bar = 10 µm. **E** Anterior hook base with fine spines (arrow) (SPL1165-2). Scale bar = 25 µm. **F** Buccal capsule (SPL1156-1). Scale bar = 100 µm. **G** SEM of male genital aperture with sensory papillae #4 on lateral edges (SPL1165-3). **H** SEM of tip of copulatory spicule emerging from male genital aperture (SPL1165-2). **I** Dissected copulatory spicule (SPL1165-1). Scale bar = 100 µm

The specimens collected from roe deer were determined to be mature adult male specimens of a species of *Linguatula* based on morphology (Figs. 3, 4). Measurements of individual specimens are provided in Table 3, compared to the original measurements of *L. arctica*, the only other species reported as an adult from an ungulate (Riley et al. 1987). The specimens in our study were determined not to be *L. serrata* based on the following characteristics: the absence of an annulus between the pair of hooks (compared to *L. serrata* which has a partial annulus that runs between), the position of the male genital opening on the second annulus (compared to *L. serrata* where it is located on the fifth annulus), and the presence of fine spines on the proximal part of the hook blades and the lining of the hook pits (compared to *L. serrata* where these have not been reported previously). Variations in the measurements of the specimens collected from deer, compared to those of *L. arctica* from reindeer, in combination with the inconclusive molecular results (see below), prevent identification of these specimens to species level. These specimens are designated as *Linguatula* sp.

**Table 3:** Morphological measurements of individual specimens of *Linguatula * sp. collected from *Capreolus capreolus* (Linnaeus) from Romania in this study compared with measurements from the literature. Measurements are presented in µm

Specimen number	1165–1	1165–2		
Characteristic	Male	Male	Male (*n* = 4)	Female
Host species	*Capreolus capreolus*	*Capreolus capreolus*	*Rangifer tarandus*	*Rangifer tarandus*
Total length	1800	1950	3400 (3200–4000)	5500–14,500
Max width	450	400		1300–2000
No. annuli	78 + ^a^	76	60 (57–62) Complete annuli + 3 incomplete	64–66
Anterior hook				
AC	–	–		
AD	–	–		670^b^
BC	185	210		
CD	300	285		
AB	–	–		
FL	610	670		
Posterior hook				
AC	–	250		
AD	–	450		
BC	215	185		
CD	290	280		
AB	–	150		
FL	660	730		
Buccal cadre L	360	415		
Buccal cadre W	300	–		
Copulatory spicule L	820 (800–840)	–	1750	
Copulatory spicule W	360 (330–390)	–		
Geographical location	Romania	Romania	Norway	Norway
Reference	This study	This study	Riley et al. (1987)	Riley et al. (1987)

^a^Specimen had been cut; number of annuli visible counted

^b^Measurement given as “hook length”

### Molecular results

All sequences obtained in the present study were deposited in GenBank under accession numbers MZ314327 to MZ314332. The 18S sequences for the sample collected from the wolf were identical to the sequences of *L. serrata* sequenced by Gjerde (2013), which was collected from a dog in Norway, originally from Romania. In addition, these sequences were identical to the Australian specimens of *L. serrata* (Shamsi et al. 2020a). The results of molecular sequencing for the *Linguatula* sp. specimen collected from the deer, however, were inconclusive, with sequences being identical to *L. serrata* for 18S. Phylogenetic analysis of 18S was not performed due to no sequence difference between *L. serrata* and *Linguatula* sp.

## Discussion

This study presents the first report of *L. serrata* in Romania through both morphological and molecular characterization. It also presents the first report of a species of *Linguatula* in roe deer, and in Romania, utilizing morphological characterization; molecular characterization was inconclusive and further work is required and will be discussed below.

Most identifications of pentastomes have been almost exclusively based on morphological and biological characteristics with only a few species subjected to molecular characterization (Kelehear et al. 2011; Gjerde 2013; Barton et al. 2019, 2020; Shamsi et al. 2020a, 2020b). Although the number of sequences of *Linguatula* spp. available in GenBank is increasing, very few of them have complementary morphological confirmation with provided morphological data, or a voucher specimen. Indeed, our study showed that the sequencing results obtained for the *Linguatula* sp. specimens were inconclusive, with a 100% match to *L. serrata*, despite clear morphological differences suggesting the 18S region of ribosomal DNA may not always be suitable for the distinction of closely related species of *Linguatula*. There is only two base pair difference (out of 1830 bp) between the 18S sequences of *L. serrata* and *L. arctica* which led Gjerde (2013) to assume any adult *Linguatula* from deer must be *L. arctica*. In addition, there are only four 18S sequences available for *L. arctica* from GenBank, all of which were published by Gjerde (2013), none with morphological details or voucher specimens. With such a low level of sequence difference, a low number of available sequences, and a lack of access to the specimens for morphological examination and comparison, it is very difficult to predict that two base pair differences are sufficient to represent species level differences in the 18S sequences; this was also suggested by Gjerde (2013). These base pair differences were absent for the *Linguatula* sp. specimen examined in this study. Whether this was due to contamination of the specimen or the sequence or another reason remains unknown. The Cox1 region, however, has shown reasonable diversity between *L. serrata* and *L. arctica* (around 10%) in other studies (Shamsi et al. 2020a, 2020b). Unfortunately, despite several attempts by us and then by other researchers, we could not obtain sequences for Cox1 from the *L. arctica* specimens in this study. Despite this inconclusive result, the authors still urge future researchers to incorporate a combined molecular and morphological approach. Recently, the sequences of 28S rRNA have shown greater suitability than the 18S region to allow for improved differentiation between species of *Linguatula* but are on par with *Cox1* (Shamsi et al. 2022). Therefore, it would be useful to investigate the suitability of 28S or other DNA regions for the reliable distinction between *Linguatula* spp.

In addition, the obvious variability in measurements of specimens of *L. serrata*, as shown in Table 2, outlines the need to undertake an extensive analysis of morphological features of specimens to determine true species boundaries. The hook measurements for the *L. serrata* specimens collected in this study were much larger than for *L. serrata* collected from dogs and foxes in Australia (Shamsi et al. 2020a). The measurements were undertaken by the same person, utilizing the same technique, which removes potential “user error.” However, the potential effect of host on the morphology of *Linguatula* spp. has not been considered. Studies on the hooks of monogenean parasites of fish have shown some relationship to either geographical location or host size (Rohde 1991; Perera 1992). Differences in the dimensions of the cranial morphology of the hosts (Morey 1992) could also be impacting the overall development of *Linguatula* specimens. A more detailed examination of the morphology of the hooks of *Linguatula* spp. in relation to their hosts and location needs to be undertaken.

*Linguatula serrata* has previously been reported from wolves in Romania (Gherman et al. 2002) as well as neighboring areas of the Balkans (Pavlović et al. 2017) and Greece (Diakou et al. 2014; Liatis et al. 2017). Gherman et al. (2002) reported 42.8% of wolves to be infected, compared to 17.5% in this study; the wolves examined by them were all collected from the same geographical area in central Romania, compared to the much wider geographical distribution examined in this study. These studies have not been able to cleave the heads of the wolves examined (as they were retained as trophies by the hunters), as was done in the study by Shamsi et al. (2017a) and thus, may underestimate the true levels of infection.

*Linguatula serrata* is also commonly reported in dogs within the same regions of Romania (Negrea et al. 2009) and the Balkans (Pavlović et al. 2017). Pavlović et al. (2017) reported that the prevalence of infection of *L. serrata* in dogs in urban areas of the Balkans had dramatically reduced from the 1930s to much lower levels now. No reasons were presented for this reduction in infection, although it is possible that changes in food source (from raw meat to commercially produced food) and/or increased urbanization may have impacted transmission of the parasite to dogs. About 5% of domestic dogs were found to be infected in Romania in 2002; Ioniță et al. (2016) reported infection of *L. serrata* in a rescued dog within the city of Bucharest, Romania. Over recent years, many stray and rescued dogs have been exported from Romania to other parts of Europe, with almost half of the 44,500 dogs imported into the UK in 2019 originating from Romania (Wright et al. 2020). A number of these dogs have been reported infected with *Linguatula* specimens, following importation to the UK (Villedieu et al. 2017; Wright et al. 2020). Indeed, the specimen of *L. serrata* sequenced by Gjerde (2013) originated from a dog that had been imported to Norway from Romania. Ioniță et al. (2016) suggested that their dog, which was approximately 6 months old, had an early infection due to the lack of pentastome eggs detected in fecal observations. However, the photograph of the specimens supplied by Ioniță et al. (2016) suggests that the pentastomes were adult females, with the pronounced brown uterus obvious. Sinclair (1954) found that *L. serrata* released eggs sporadically following an experimental infection of dogs, and it may be that Ioniță et al. (2016) were unable to detect infection due to this. This suggests that surveillance of imported dogs for infection of *L. serrata* needs to be undertaken across a number of fecal examinations over a period.

*Linguatula arctica*, reported as an adult from an ungulate, was originally described by Riley et al. (1987) from ten specimens (four males, six females) collected from semi-domesticated reindeer in Norway. In the description of the new species, body measurements (length, width, and annulus count) were provided, but hook measurements were only provided for one female specimen, with an overall length of 670 µm. Morphological criteria suggested to help identify *L. arctica* included very large female body size (14.5 cm), which is sharply delineated between the broad anterior section and the thin “tail,” the low number of annuli (at the time of description, the lowest number of all the species of *Linguatula*), and the extensive patch of minute spines covering the posterior hook. For the male specimens described by Riley et al. (1987), the posterior hook was not covered in minute spines, although they were present in the hook pit, and there was a row of small spines along the posterior margin of the body annuli. The genital opening for the male *L. arctica* was found on the second annulus and there did not appear to be a defined annulus running between the anterior and posterior pair of hooks, similar to *L. nuttalli* Sambon, 1922 (Shamsi et al. 2020b) compared to *L. serrata* (Shamsi et al. 2020a). The specimens collected from deer in this study were very similar in overall morphology to *L. arctica*. However, with the low number of specimens available, the differences in measurements and the new host and geographical location, it was determined to identify the specimens as *Linguatula* sp. pending further research.

The presence of the spines in the hook pit of specimens of *Linguatula* needs to be examined further. Although listed as a diagnostic feature in *L. arctica*, as described above, they have never been mentioned for other species of *Linguatula*. However, the SEM of the adult male *L. serrata* collected from the wolf in this study (Fig. 2) clearly showed spines lining the hook pit. An examination of SEMs presented in earlier published works does not show such spines; however, most specimens also do not have a clear view of the hook pit. The presence of the spines was also observed on light microscopy in this study. Thus, future work should dissect hooks from adults to determine the presence of these spines and their potential importance in species delineation.

Riley et al. (1987) found infection of *L. arctica* to predominate in younger reindeer, with 60% of 48 reindeer (under 6 months of age) infected and decreasing in prevalence with no reindeer aged over 28 months infected. Riley et al. (1987) also listed other reports of *L. arctica* from reindeer, and its various subspecies, from around the Arctic Circle, which had originally been ascribed to infection with *L. serrata*. Further studies have reported *L. arctica* in reindeer from Norway (Haugerud et al. 1990; Gjerde 2013) and Finnish Lapland (Nikander and Saari 2009). Although these reports do not include morphological measurements, Nikander et al. (2009) showed the structure of the small spines that covered the proximal parts of the hooks in both female and male specimens.

Roe deer are native to Romania (Ionescu et al. 2010), with few studies documenting their parasite fauna in this region of Europe. Hora et al. (2017) surveyed 122 deer across three species and found a number of nematodes, including *Dictyocaulus capreolus* Gibbons and Höglund, 2002 in the lungs of 26% of 73 roe deer. However, no mention of other parasites in the lungs was reported. Shimalov (2002) examined 16 roe deer in Belarus and did not report any *Linguatula*.

At the time of collection of the pentastomes from the lungs of the deer, due to their location, it was thought that they were nymphs of *L. serrata*; subsequent morphological examination determined that they were in fact adult male *Linguatula* sp. Adult male pentastomes are similar to nymphs in morphology, being much smaller than the female. Also, the finding of them not in a nasal sinus and in a host that has not been recorded as a definitive host previously, could suggest that the specimens were nymphs. Dependent on how long the host animal had been dead, the pentastomes may have migrated from the nasal cavities to the lungs or, given the smaller overall size of the *Linguatula* sp. specimens in this study compared to the measurements provided by Riley et al. (1987), it may be that they were not yet in their final location of infection. It may be possible that the life cycle in ungulates involves development within the lung, or other internal organs, as in the life cycle of *L. serrata*, before migration to the nasal sinus, given the truncated life cycle as postulated by Haugerud (1989) and Haugerud et al. (1990). This highlights the need to examine all specimens of *Linguatula* to ensure that they are, in fact, nymphs, not adults, and not to disregard the fact that adult *Linguatula* species occur in hosts other than canids. *Linguatula arctica*, itself, was mistakenly identified as *L. serrata*, despite being found in a number of ungulates by many authors (Riley et al. 1987). Riley et al. (1987) provided a list of reports of *Linguatula* collected from various ungulates and emphasized the need to carefully examine wild ungulates in other parts of the world. Until this study, there have been no other reports of *Linguatula* infections in other groups of ungulates. However, a list of the early reports of *L. serrata*, under its various names, Poore (2012a, 2012b) does include the original description by Chabert (1787) with specimens collected from the sinus of a horse. Shipley (1898) also stated that adult *L. serrata* were found in the frontal sinus of horses, mules, and sheep, while Christoffersen and De Assis (2013) list all reports of *L. serrata* infections, including reports from the lungs of a bull and the sinus of sheep. Haugerud (1989) postulates that these infections in livestock species are rare and accidental, although he also suggests that it may be a new species of *Linguatula* or a facultative life cycle strategy. Either way, although nymphal *L. serrata* have been reported from the lungs of various animals (Liatis et al. 2017; Barton et al. 2019, 2020), the presence of pentastomids in this organ should not be immediately referred to as a nymphal *L. serrata* without morphological verification.

## References

[CR1] Barton DP, Porter M, Baker A, Zhu X, Jenkins DJ, Shamsi S (2019). First report of nymphs of the introduced pentastomid, *Linguatula serrata*, in red-necked wallabies (*Notamacropus rufogriseus*) in Australia. Aust J Zool.

[CR2] Barton DP, Baker A, Porter M, Zhu X, Jenkins D, Shamsi S (2020) Verification of rabbits as intermediate hosts for *Linguatula serrata* (Pentastomida) in Australia. Parasitol Res 119(5):1553–1562. 10.1007/s00436-020-06670-y 10.1007/s00436-020-06670-y 32236709

[CR3] Berberich M, Grochow T, Roßner N, Schmäschke R, Rentería-Solís Z (2022). *Linguatula serrata* in an imported dog in Germany: single-case or emerging disease?. Veterinary Parasitology: Regional Studies and Reports.

[CR4] Chabert P (1787) Traité des maladies vermineuses dans les animaux. de l'Imprimerie Royale

[CR5] Christoffersen ML, De Assis JE (2013). A systematic monograph of the recent Pentastomida, with a compilation of their hosts. Zoologische Mededelingen.

[CR6] Diakou A, Karaiosif R, Petridou M, Iliopoulos Y (2014) Endoparasites of the wolf (*Canis lupus*) in central Greece. Poster No 113, EWDA 2014–11th European Wildlife Disease Association Conference

[CR7] Frölich JAv (1789) Beschreibungen einiger neuen Eingeweidewürmer. Der Naturforscher 24:101–162

[CR8] Gherman C, Cozma V, Mircean V, Brudasca F, Rus N, Detasan A (2002). Helminthic zoonoses in wild carnivours species from Romanian fauna. Scient Parasitol.

[CR9] Gjerde B (2013). Phylogenetic position of *Linguatula arctica* and *Linguatula serrata* (Pentastomida) as inferred from the nuclear 18S rRNA gene and the mitochondrial cytochrome c oxidase subunit I gene. Parasitol Res.

[CR10] Haugerud R (1989). Evolution in the pentastomids. Parasitol Today.

[CR11] Haugerud RE, Nilssen AC (1990). Life history of the reindeer sinus worm, *Linguatula arctica* (Pentastomida), a prevalent parasite in reindeer calves. Rangifer Special Issue.

[CR12] Hora F, Genchi C, Ferrari N, Morariu S, Mederle N, Dărăbuș G (2017). Frequency of gastrointestinal and pulmonary helminth infections in wild deer from western Romania. Veterinary Parasitology: Regional Studies and Reports.

[CR13] Ionescu O, Macinic C, Lazar R. The dynamics of deer species in Cris Forest District and the influence of fallow deer (*Dama dama* L.) dynamic on red deer (*Cervus elaphus* L.) and roe deer (*Capreolus capreolus* L.) populations. Proceedings of the biennial international symposium, forest and sustainable development, Brașov, Romania, 15–16th October 2010. Transilvania University Press, p 339–344

[CR14] Ioniță M, Mitrea IL (2016). *Linguatula serrata* (Pentastomida: Linguatulidae) infection in dog, Romania: a case report. AgroLife Sci J.

[CR15] Kearse M, Moir R, Wilson A, Stones-Havas S, Cheung M, Sturrock S, Buxton S, Cooper A, Markowitz S, Duran C, Thierer T, Ashton B, Meintjes P, Drummond A (2012). Geneious Basic: an integrated and extendable desktop software platform for the organization and analysis of sequence data. Bioinformatics.

[CR16] Kelehear C, Spratt DM, Dubey S, Brown GP, Shine R (2011) Using combined morphological, allometric and molecular approaches to identify species of the genus *Raillietiella* (Pentastomida). PLoS ONE 6(9) 10.1371/journal.pone.0024936 10.1371/journal.pone.0024936 PMC3176809 21949796

[CR17] Liatis TK, Monastiridis AA, Birlis P, Prousali S, Diakou A (2017). Endoparasites of wild mammals sheltered in wildlife hospitals and rehabilitation centres in Greece. Frontiers in Veterinary Science.

[CR18] Morey DF (1992). Size, shape and development in the evolution of the domestic dog. J Archaeol Sci.

[CR19] Negrea O, Liviu O, Miclaus V, Miresan V, Răducu C, Marchis Z (2009) Diagnosis epidemiologic observations in dog linguatulosis. Lucrări Științifice-Medicină Veterinară, Universitatea de Științe Agricole și Medicină Veterinară" Ion Ionescu de la Brad" Iași 52(11 (2)):694–697

[CR20] Nikander S, Saari S (2009). A SEM study of the reindeer sinus worm (*Linguatula arctica*). Rangifer.

[CR21] Pavlović I, Penezić A, Ćosić N, Burazerović J, Maletić V, Ćirović D (2017). The first report of *Linguatula serrata* in grey wolf (*Canis lupus*) from Central Balkans. Journal of the Hellenic Veterinary Medical Society.

[CR22] Perera KML (1992). The effect of host size on large hamuli length of *Kuhnia scombri* (Monogenea: Polyopisthocotylea) from Eden, New South Wales. Australia Inte J Parasit.

[CR23] Poore GCB (2012). The nomenclature of the recent Pentastomida (Crustacea), with a list of species and available names. Syst Parasitol.

[CR24] Poore GCB (2012). The nomenclature of the recent Pentastomida (Crustacea), with a list of species and available names (vol 82, pg 211, 2012). Syst Parasitol.

[CR25] Riley J, Haugerud RE, Nilssen AC (1987). A new species of pentastomid from the nasal passages of the reindeer (*Rangifer tarandus*) in northern Norway, with speculation about its life-cycle. J Nat Hist.

[CR26] Rohde K (1991). Size differences in hamuli of *Kuhnia scombri* (Monogenea: Polyopisthocotylea) from different geographical areas not due to differences in host size. Int J Parasitol.

[CR27] Shamsi S, Briand MJ, Justine J-L (2017). Occurrence of *Anisakis* (Nematoda: Anisakidae) larvae in unusual hosts in Southern Hemisphere. Parasitol Int.

[CR28] Shamsi S, McSpadden K, Baker S, Jenkins DJ (2017b) Occurrence of tongue worm, *Linguatula cf. serrata* (Pentastomida: Linguatulidae) in wild canids and livestock in south-eastern Australia. International Journal for Parasitology: Parasites and Wildlife 6(3):271–277 10.1016/j.ijppaw.2017.08.008 10.1016/j.ijppaw.2017.08.008 PMC5604946 28971014

[CR29] Shamsi S, Barton DP, Zhu X, Jenkins DJ (2020). Characterisation of the tongue worm, *Linguatula serrata* (Pentastomida: Linguatulidae), in Australia. International Journal for Parasitology: Parasites and Wildlife.

[CR30] Shamsi S, Halajian A, Barton DP, Zhu X, Smit WJ, Roux F, Luus-Powell WJ (2020). Occurrence and characterisation of tongue worms, *Linguatula* spp., in South Africa. International Journal for Parasitology: Parasites and Wildlife.

[CR31] Shamsi S, Zhu X, Halajian A, Barton DP (2022) 28S rRNA sequences for *Linguatula* spp. Parasitol Res 121(6):1799–1804. 10.1007/s00436-022-07507-6 10.1007/s00436-022-07507-6 PMC9098581 35362745

[CR32] Shimalov V (2002). Helminth fauna of cervids in Belorussian Polesie. Parasitol Res.

[CR33] Shipley AE (1898) An attempt to revise the family Linguatulidae. Kessinger Publishing, Llc,

[CR34] Sinclair K (1954). The incidence and life cycle of *Linguatula serrata* (Frohlich 1789) in Great Britain. Journal of Comparative Pathology and Therapeutics.

[CR35] Villedieu E, Sanchez RF, Jepson RE, Ter Haar G (2017). Nasal infestation by *Linguatula serrata* in a dog in the UK: a case report. J Small Anim Pract.

[CR36] Wright I, Collins M, McGarry J, Teodoru S, Constantin SA, Corfield EL, Harding I (2020). Threat of exotic worms in dogs imported from Romania. Veterinary Record.

